# Lack of neuroprotection in the absence of P2X7 receptors in toxin-induced animal models of Parkinson's disease

**DOI:** 10.1186/1750-1326-6-28

**Published:** 2011-05-04

**Authors:** Zsuzsanna Hracskó, Mária Baranyi, Cecilia Csölle, Flóra Gölöncsér, Emilia Madarász, Ágnes Kittel, Beáta Sperlágh

**Affiliations:** 1 Laboratory of Molecular Pharmacology, Institute of Experimental Medicine, Hungarian Academy of Sciences, H-1083 Budapest, Szigony u. 43, Hungary; 2 Laboratory of Cellular and Developmental Neurobiology, Institute of Experimental Medicine, Hungarian Academy of Sciences, H-1083 Budapest, Szigony u. 43, Hungary

## Abstract

**Background:**

Previous studies indicate a role of P2X_7 _receptors in processes that lead to neuronal death. The main objective of our study was to examine whether genetic deletion or pharmacological blockade of P2X_7 _receptors influenced dopaminergic cell death in various models of Parkinson's disease (PD).

**Results:**

mRNA encoding P2X_7 _and P2X_4 _receptors was up-regulated after treatment of PC12 cells with 1-methyl-4-phenyl-1,2,3,6- tetrahydropyridine (MPTP). P2X_7 _antagonists protected against MPTP and rotenone induced toxicity in the LDH assay, but failed to protect after rotenone treatment in the MTT assay in PC12 cells and in primary midbrain culture. *In vivo *MPTP and *in vitro *rotenone pretreatments increased the mRNA expression of P2X_7 _receptors in the striatum and substantia nigra of wild-type mice. Basal mRNA expression of P2X_4 _receptors was higher in P2X_7 _knockout mice and was further up-regulated by MPTP treatment. Genetic deletion or pharmacological inhibition of P2X_7 _receptors did not change survival rate or depletion of striatal endogenous dopamine (DA) content after *in vivo *MPTP or *in vitro *rotenone treatment. However, depletion of norepinephrine was significant after MPTP treatment only in P2X_7 _knockout mice. The basal ATP content was higher in the substantia nigra of wild-type mice, but the ADP level was lower. Rotenone treatment elicited a similar reduction in ATP content in the substantia nigra of both genotypes, whereas reduction of ATP was more pronounced after rotenone treatment in striatal slices of P2X_7 _deficient mice. Although the endogenous amino acid content remained unchanged, the level of the endocannabinoid, 2-AG, was elevated by rotenone in the striatum of wild-type mice, an effect that was absent in mice deficient in P2X_7 _receptors.

**Conclusions:**

We conclude that P2X_7 _receptor deficiency or inhibition does not support the survival of dopaminergic neurons in an *in vivo *or *in vitro *models of PD.

## Background

The role of extracellular ATP and purinoceptors in neurodegeneration is the focus of a promising area of research [1-3]. P2X_7 _receptors belong to the ionotropic P2X receptors [4], which are sensitive to ATP and other nucleotides. The homo-oligomeric P2X_7 _receptor has distinct structural, functional and pharmacological features within the P2X receptor family [2,3,5]: (1) its intracellular carboxy terminal domain is longer than the other P2X receptor subunits; (2) it has several splice variants that display distinct functionalities in P2X_7 _receptor wild-type and knockout mice [6]; (3) its persistent activation elicits the opening of a membrane pore, which eventually leads to cell death in certain cell types; and (4) it needs high micromolar concentrations of ATP to be activated.

P2X_7 _receptors probably affect neuronal death and survival in a complex way. An important role of P2X_7 _receptors in the brain is the modulation of neurotransmitter release [3,7]. The activation of P2X_7 _receptors elicits Ca^2+ ^influx, which is followed by increased glutamate and subsequent GABA release [8-10] that could reinforce glutamate-mediated excitotoxicity under pathological conditions. In addition, P2X_7 _receptors play a governing role in the activation and proliferation of microglia following pathological signals [11,12] and directly contribute to neurodegeneration by eliciting microglia-mediated neuronal death [13]. Moreover, P2X_7 _receptors are involved in the processing and release of other key mediators in neurodegeneration, such as interleukin-1β, [2,14], and in the production of endocannabinoids, which are neuroprotective. In fact, P2X_7 _receptor activation is one of the most powerful stimuli that lead to the synthesis and subsequent release of endocannabinoids from activated microglia [15] and astrocytes [16].

Accordingly, the blockade or absence of P2X_7 _receptors were found to be neuroprotective in animal models of spinal cord injury [17,18], Alzheimer's disease (AD) [19] and Huntington's disease [20], but conflicting results have been found in animal models of ischemia-reperfusion ([21,22] but see [23,24]) and multiple sclerosis ([25] but see [26,27]). However, data regarding P2X_7 _receptors in the striatum and its role in PD are scarce. In dopaminergic co-cultures, PCR data indicate the expression of P2X_7 _mRNA in neonatal and adult rats [28], and the presence of P2X_7 _receptor protein has also identified in the rat brain striatum and substantia nigra [29]. Functional P2X_7 _receptors have been detected in synaptosomal preparations from the rat midbrain [30] and in organotypic striatal cultures [31]. In SN4741 dopaminergic cells, ATP-induced swelling and cell death are reversed by the P2X_7 _receptor antagonist, KN62, or by the knock-down of P2X_7 _receptors with small interfering RNAs [32]. Nevertheless, evidence of a protective or aggravating role of P2X_7 _receptors in an *in vivo *animal model of PD is still lacking.

This work provides insight on the effect of P2X_7 _receptor deficiency in different models of PD. The mRNA encoding P2X_7 _receptors was up-regulated after rotenone or MPTP treatment in PC12 cells and in the striatum, but these changes were not translated to P2X_7 _receptor protein expression in the striatum. Although P2X_7 _receptor antagonists displayed some protective effect against rotenone-induced toxicity in PC12 cells, these data could not be replicated in primary dopaminergic neurons, in MPTP-induced toxicity or in an *in vitro *and an *in vivo *animal model of PD.

## Results

### Effect of *in vitro *MPTP treatment on the expression of P2X_7 _and P2X_4 _receptors

**Figure 1:**
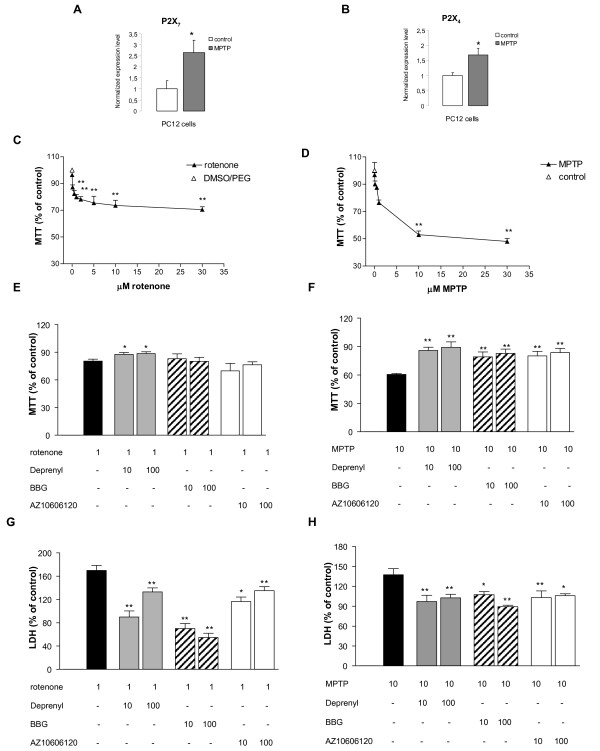
**Effect of P2X_7 _receptor antagonists on cell viability after rotenone and MPTP pretreatment in PC12 cells**. A. and B. Expression levels of the P2X_7 _(A) and P2X_4 _(B) purinergic receptors in PC12 cells after 1 μM MPTP treatment. Quantitative SYBR Green real-time PCR was performed using specific primers. The experiments were repeated twice with similar results. The expression level of the P2X receptors was normalized to the expression level of the distinct housekeeping gene, 18S rRNA. Data are displayed as the mean ± S.E.M. Asterisks indicate significant differences from the corresponding control (*P < 0.05, Student's t-test). C and D. Concentration-dependent inhibition of cell viability by rotenone (C) and MPTP (D) in the MTT assay. Cells were treated with various concentrations of the toxins ranging from 0.01 μM to 30 μM, and the reduction of MTT into formazan was measured 20 h later. Cell viability is expressed as a percentage of the respective controls. E and F. Effects of BBG and AZ10606120 on toxicity induced by rotenone (E) and MPTP (F) measured in the MTT assay. Cells were pretreated with L-deprenyl and with the P2X_7 _antagonists, BBG and AZ10606120, in 10 and 100 nM concentrations for 1 h before treatment with 1 μM rotenone or 10 μM MPTP for 20 h. Data are expressed as the percentage of values of control cultures and are the means ± S.E.M. of four experiments. G. and H. Effects of BBG and AZ10606120 on toxicity induced by rotenone (G) and MPTP (H) measured in the LDH assay. Treatments of the cells were identical to the MTT assay. The released LDH is expressed as the percentage of total LDH measured after freeze/thaw lysis of cells. These data are then expressed as the percentage of values of control cultures and are the means ± S.E.M. of four experiments. * *P *< 0.05, ** *P *< 0.01, significance vs. controls using an ANOVA followed by the Dunnett test. E-H. Note that the concentration of rotenone and MPTP are indicated in μM, whereas the concentration of other drugs in nM.

Changes in the levels of mRNA transcripts of the P2X_7 _and P2X_4 _receptor subunits were measured using real-time RT-PCR in PC12 cells after 24 h of a 1 μM *in vitro *MPTP treatment (Figures 1A, B). Gene expression level was normalized to the expression level of the 18S rRNA reference gene. Quantitative real-time PCR measurements revealed that the mRNA expression of the P2X_7 _receptor was up-regulated to 2.65 ± 0.55 of the corresponding control values, established as 1 and normalized to 18S rRNA (Figure 1A; n = 5, *P *< 0.05). Likewise, the expression level of the P2X_4 _receptor was up-regulated to 1.69 ± 0.22 of the corresponding control values (Figure 1B; *n *= 5, *P *< 0.05).

### Effect of P2X_7 _receptor antagonists on rotenone and MPTP-induced inhibition of cell viability in PC12 cells

In conjunction with our previous study [33], treatment of PC12 cells with the mitochondrial complex I inhibitor, rotenone, elicited a concentration-dependent (0.01-30 μM) decline in cell viability, measured by the MTT assay, that resulted in a significant decrease to 79.2 ± 3.7% of the control after a 20 h treatment with a 1 μM concentration (n = 4, P < 0.01, Figures 1C, E). The same treatment elevated the amount of released LDH, another indicator of cellular death, to 169.71 ± 8.91% (n = 4, P < 0.01) (Figure 1G). The treatment of the cells with MPTP also concentration-dependently (0.01-30 μM) decreased cell viability in the MTT assay (Figure 1D). The decrease in cell survival reached a significant level at 10 μM concentration (60.68 ± 1.07%, n = 40, P < 0.01, Figures 1D, F). This treatment also elicited a significant increase in the amount of released LDH (137.42 ± 9.08%, n = 8, P < 0.01, Figure 1H). The MAO-B inhibitor, L-deprenyl (10-100 nM), displayed significant protection against the decrease in cell viability elicited by rotenone measured either by using the MTT (Figure 1E) or the LDH assays (Figure 1G). A consistent protective effect was also observed after MPTP treatment (Figures 1F, H). Pretreatment with the selective P2X_7 _receptor antagonists, BBG (10 and 100 nM) and AZ10606120 (10 and 100 nM) had no effect on cell viability after rotenone treatment when measured with the MTT assay (Figure 1E). However, identical treatments with the P2X_7 _receptor antagonists significantly attenuated the effect of MPTP in the MTT assay (Figure 1F) and the effect of both rotenone (Figure 1G) and MPTP (Figure 1H) in the LDH assay.

### Effect of P2X_7 _receptor antagonists on rotenone- and MPTP-induced inhibition of cell viability in primary midbrain cultures

**Figure 2:**
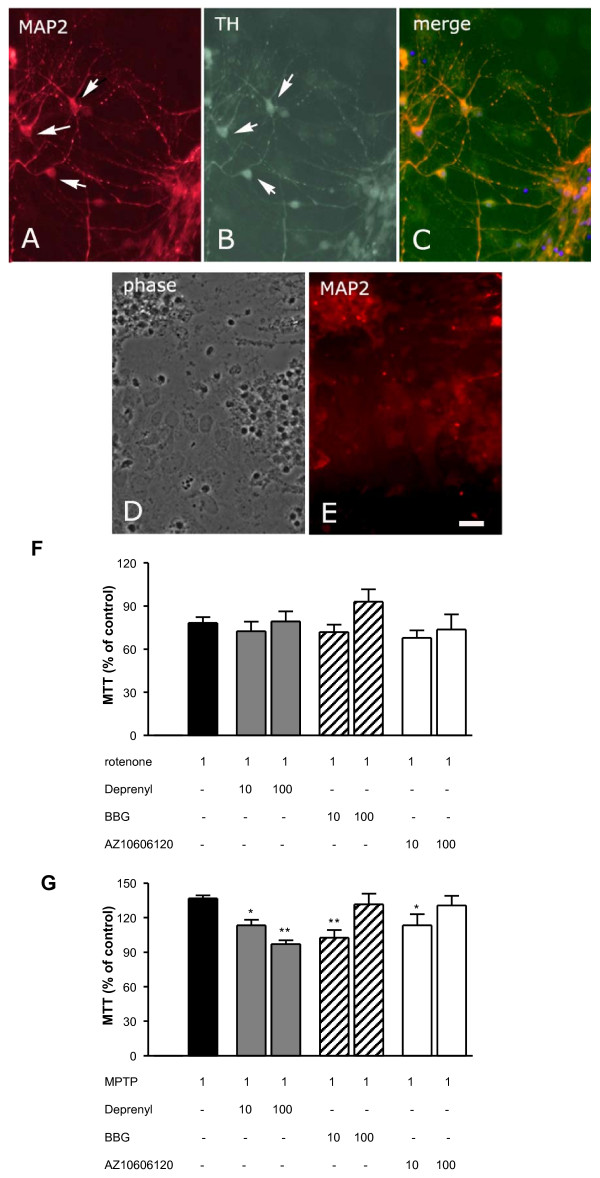
**Effect of P2X_7 _receptor antagonists on cell viability after rotenone and MPTP pretreatment in primary substantia nigra (SN) culture**. Non-treated SN cultures contained large tyrosine hydroxylase-positive (arrow) neurons that were identified by MAP2 (red; A) and TH (green; B) immunoreactivity (merge; C). Rotenone treatment disrupted the neurons (phase contrast morphology; D and MAP2 immunostaining; E). Scale bar: 20 μm. MAP2+ and TH+ neurons represented 26.28 ± 10.6% and 7.32 ± 2.81% of the total cell number. Total cell number was determined by counting DAPI-positive nuclei on each of 40 microscopic fields. Cells were counted at 250 × (2 × 10 fields) and 500 × (2 × 10 fields) magnification (independent determinations: n = 4). F. and G. Effects of BBG and AZ10606120 on changes in cell viability induced by rotenone (F) and MPTP (G) measured in the MTT assay. Cells were pretreated with L-deprenyl and with the P2X_7 _antagonists, BBG and AZ10606120, in 10 and 100 nM concentrations for 1 h before treatment with 1 μM rotenone or MPTP for 20 h. Data are expressed as the percentage of values of control cultures and are the means ± S.E.M. of four experiments. *P < 0.05, ** *P *< 0.01, significance vs. controls using an ANOVA followed by the Dunnett test. Note that the concentration of rotenone and MPTP are indicated in μM, whereas the concentration of other drugs in nM.

Because the molecular machinery of degeneration of PC12 cells and dopaminergic neurons are not necessarily the same, we next sought to determine how P2X_7 _receptor antagonists affect cell viability in primary substantia nigra cultures that contain dopaminergic neurons, which can be identified by tyrosine hydroxylase immunoreactivity. As shown in Figures 2A-C, MAP2-positive neurons displayed strong tyrosine hydroxylase immunoreactivity in the culture after 8 days *in vitro*. Similar to what we observed in PC12 cells, the treatment of cultures with rotenone (1 μM) elicited a significant decline in cell viability when measured in the MTT assay (78.24 ± 4.15%, n = 7, P = 0.05, Figure 2F). The morphological destruction of MAP2 neurons was also clearly observed (Figures 2D, E). Using this model, we did not observe any protective effect using deprenyl, BBG or AZ10606120 (Figure 2F). Interestingly, MPTP (1 μM) caused an elevation of cell viability in these experiments (136.8 ± 2.49%, n = 7, P < 0.01, Figure 2G), and L-deprenyl (10-100 nM), BBG (10 nM) and AZ10606120 (10 nM) attenuated these effects (Figure 2G).

### Effect of *in vivo *MPTP treatment on the expression of P2X_7 _and P2X_4 _purinergic receptors

**Figure 3:**
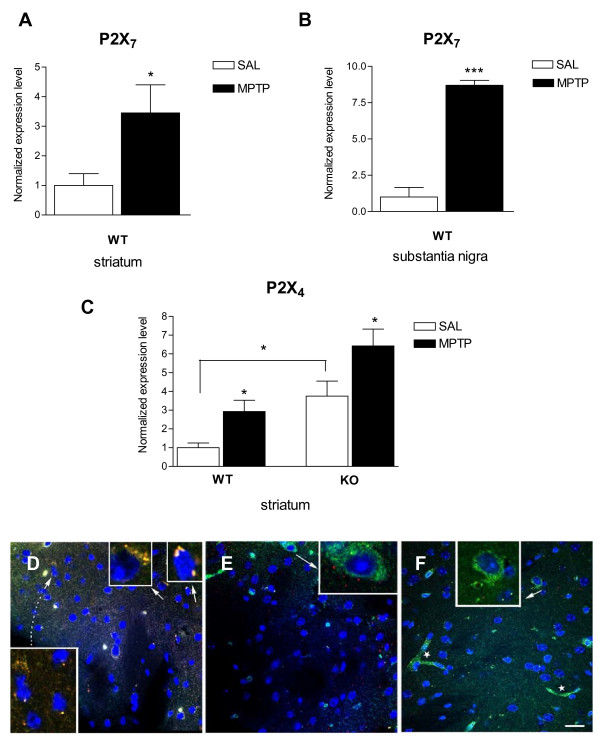
**Changes in the mRNA and protein expression of P2X_7 _and P2X_4 _receptors in the striatum and substantia nigra obtained from P2X_7 _receptor wild-type (WT) and P2X_7_-/- (KO) mice after *in vivo *MPTP treatment**. Mice (6-8 mice per group) were injected i.p. with 4 × 20 mg/kg of MPTP or saline (SAL). A-C. After decapitation, the brains were removed immediately, total RNA extracted from the striatum (A, C) and substantia nigra (B) and then reverse-transcribed to cDNA. Data are displayed as the means ± S.E.M. Asterisks indicate significant differences from the corresponding saline-injected mice or between genotypes as indicated (*P < 0.05, ***P < 0.0001). D, E. and F. Immunofluorescent staining for P2X_7 _receptor and microglial cells in striatal sections of saline- and MPTP-treated WT and P2X_7_-/- mice. Merged pictures. Fluorescein-labeled GSL I - isolectin B4 is a marker for endothelial (see stars in picture "F") and microglial cells. P2X_7 _receptors were labeled with a P2X_7_-DyLight 549 conjugate (red). Orange means the same localization for both stains. DAPI (in Vectashield mounting medium) labels nuclei (blue). Scale bar: 20 μm. D. Detail of the striatal section of saline-treated WT mouse brain. Inserts (arrows) show the localization of P2X_7 _receptors in FITC-labeled microglial cells. E. Only microglial cells are labeled (green, insert, arrow) in the striatal section of MPTP-treated WT mouse brain. F. Microglia (arrow, insert) and endothelial cells (stars) are labeled by the isolectin, but no P2X_7 _immunofluorescence is visible in the striatal sections of saline-treated P2X_7_-/- mouse brain.

Similar to what was seen after *in vitro *MPTP treatment, the *in vivo *treatment of WT mice with the dopaminergic neurotoxin, MPTP (4 × 20 mg/kg i.p.), caused an up-regulation of the mRNA levels of the P2X_7 _receptor in the striatum to 3.45 ± 0.95 of the corresponding control values (Figure 3A, n = 8, *P *< 0.05). Likewise, mRNA encoding P2X_7 _receptor was strongly up-regulated to 8.70 ± 0.34 of the corresponding control values, established as 1, normalized to 18S rRNA in the substantia nigra (Figure 3B, n = 4, P < 0.0001). The expression level of the P2X_4 _receptor was up-regulated both in WT and P2X_7_-/- mice to 2.93 ± 0.6 and 6.42 ± 0.9 of the corresponding control values (Figure 3C, n = 6, *P *< 0.05). It is noteworthy that the basal expression of the P2X_4 _receptor in P2X_7_-/- mice was also significantly elevated (3.75 ± 0.8, n = 6, *P *< 0.05) compared to the basal expression level of the WT group (Figure 3C). When the expression of P2X_7 _receptors in the striatum was examined at the protein level using a specific antibody, a relatively weak P2X_7 _receptor immunoreactivity was found in the striatum, in agreement with a previous study in rats [29]. Punctate immunostaining was observed throughout the striatum and was co-localized to microglial cells, which were identified by GSL I - isolectin B4 marker in saline-treated WT mice (Figure 3D). No apparent change in the expression of P2X_7 _receptor was visible in striatal slices derived from WT mice that had undergone *in vivo *MPTP treatment; if any, only a slight decrease in its expression was observed (Figure 3E). The staining intensity of P2X_7 _receptor immunoreactivity was identical with the background in striatal slices prepared from P2X_7_-/- mice treated with saline (Figure 3F).

### Effect of *in vivo *MPTP treatment on biogenic amine content of the striatum

**Figure 4:**
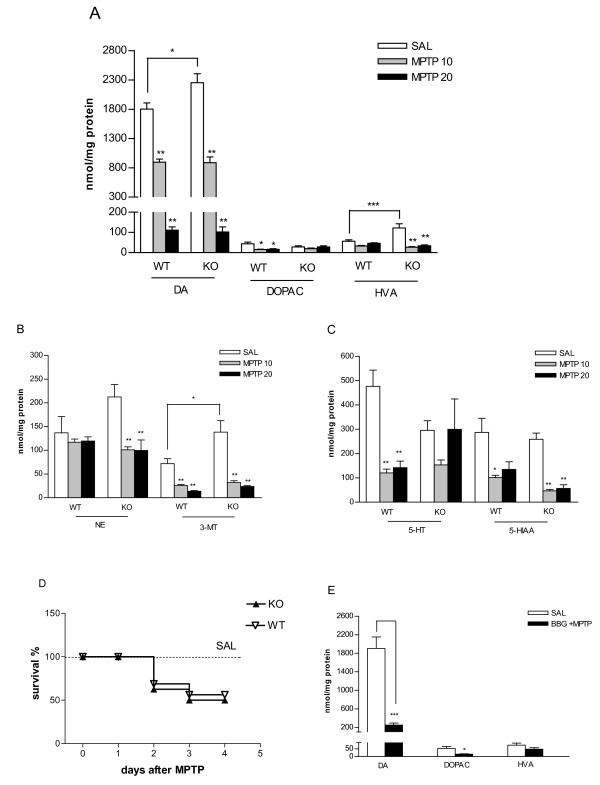
**Effect of genetic deletion and pharmacological antagonists of P2X_7 _receptors on the level of DA and its metabolites in the striatum and animal survival after *in vivo *MPTP treatment**. Wild-type (WT) and P2X_7 _-/- (KO) mice were treated with saline or with MPTP in doses indicated in the legend (4 × 10-20 mg/kg i. p.) and sacrificed 72 h later. A, B, C and E. Striatal samples were analyzed using HPLC and the amount of (A, E) dopamine (DA) 3,4-dihydroxyphenylacetic acid (DOPAC), homovanillic acid (HVA), (B) norepinephrine (NE), 3-methoxityramine (3-MT) and (C) 5-HT and 5-hydroxyindoleacetic acid (5-HIAA) is expressed in nmol/mg protein (n = 5-8). D. Survival curves of WT and P2X_7 _-/- (KO) mice subjected to *in vivo *MPTP (4 × 20 mg/kg i.p.) treatment (n = 16/group). E. Effect of Brilliant blue G (BBG, 50 mg/kg i.p.) pretreatment on the level of DA and its metabolites in the striatum of WT mice after *in vivo *MPTP (4 × 20 mg/kg) treatment. BBG was injected 18 h before the first MPTP injection. Asterisks indicate significant differences from the corresponding saline-injected mice or between WT and KO mice as indicated (*P < 0.05, ** *P *< 0.01, *** *P *< 0.01).

HPLC analysis of the tissue content of DA and its metabolites, DOPAC, HVA, norepinephrine (NE) and 3-methoxytyramine (3-MT), and that of serotonin (5-HT) and 5-hydroxyindoleacetic acid (5-HIAA) showed that, in saline-treated animals, DA, HVA and 3-MT levels were significantly higher in mice genetically deficient in P2X_7 _receptors compared to WT mice (Figures 4A, B). We then examined how MPTP treatment (4 x10-20 mg/kg i.p.) in WT and P2X_7_-/- mice changed the levels of these compounds. MPTP caused a dose-dependent and profound depletion of DA content in the WT group, which represents a biochemical feature of PD. Similar to DA, there was a significant decline in the amount of DA metabolites (e.g., DOPAC, 3-MT) in the WT group, but no significant decline in the amount of NE was observed (Figures 4A, B). In the P2X_7_-/- mice, MPTP treatment elicited a similar reduction of endogenous DA content as the WT mice (Figure 4A). Moreover, in the P2X_7_-/- group, the reduction of NE, HVA and 3-MT levels after MPTP treatment was significant, and these changes were manifest at the lower dose of MPTP treatment (Figures 4A, B). In contrast, the content of DOPAC did not change after MPTP treatment in P2X_7 _receptor deficient animals (Figure 4B). 5-HT and its metabolite (5-HIAA) were decreased in the WT mice, whereas in the P2X_7_-/- group, 5-HT levels were preserved after MPTP treatment and only 5-HIAA was depleted (Figure 4C).

Treatment with the higher dose (4 × 20 mg/kg) of MPTP elicited a significant reduction of the survival of WT mice at the 72 h endpoint of the study (9 out of 16 animals, 56.25%, P < 0.01) compared to saline-treated animals (13 out of 13 animals, 100%). Genetic deletion of P2X_7 _receptors alone did not change mortality in the saline-treated group (10 out of 10 animals, 100%). A decrease in survival after MPTP (4 × 20 mg/kg) treatment was also evident in P2X_7_-/- mice (8 out of 16 animals, 50%, P = 0.03), but this effect was not significantly different from the WT group (P > 0.05, Figure 4D). The lower dose of MPTP treatment (4 × 10 mg/kg i.p.) did not change the survival rate either in WT (6 out of 6 animals, 100%) or P2X_7_-/- mice (6 out of 6 animals, 100%).

When mice were pretreated with the P2X_7 _receptor antagonist BBG in a dose previously shown to be protective in animal models of HD [20] and spinal cord injury [17], endogenous DA levels were depleted by MPTP compared to saline-treated mice (Fig. 4E) similar to what was observed in mice treated only with MPTP (Figure 4A). Likewise, a reduction of DOPAC levels was also observed in mice pretreated with BBG 18 h before MPTP treatment (Figure 4E).

### *In vitro *rotenone treatment

**Figure 5:**
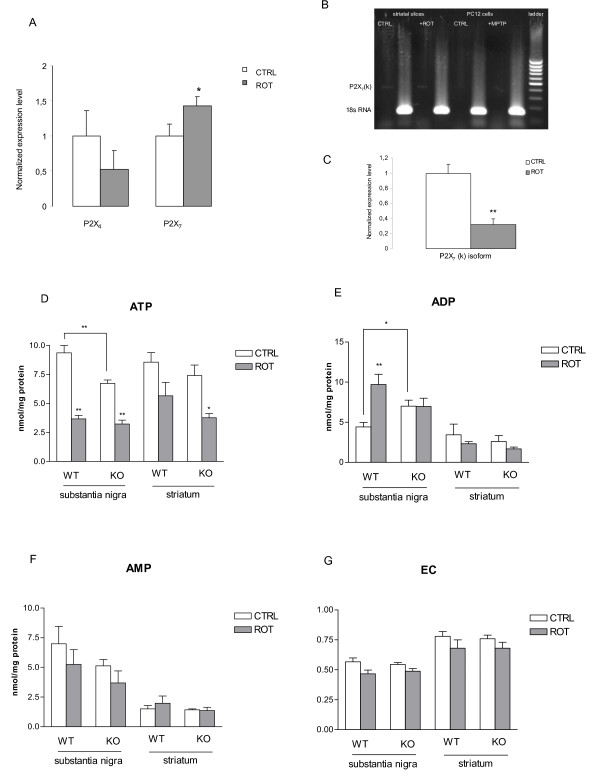
**The effect of *in vitro *rotenone pretreatment on the mRNA expression of P2X_7 _and P2X_4 _receptors and the P2X_7 _(k) splice variant, ATP, ADP and AMP contents and on the energy charge in the substantia nigra and striatal slices of wild-type (WT) and P2X_7 _receptor knockout (KO) mice**. Striatal slices were preincubated with *in vitro *rotenone (10 μM) for 60 min. (A) Quantitative SYBR Green real-time PCR was performed using specific primers. The experiments were repeated twice with similar results. The expression level of the P2X receptors was normalized to the expression level of the distinct housekeeping gene, 18S rRNA. Data are displayed as the means ± S.E.M. Asterisks indicate significant differences from the corresponding control (*P < 0.05, Student's t-test). B. Confirmation of the expression of the P2X_7_(k) variant using RT-PCR with specific forward primers for P2X_7_(k) isoforms and a common reverse primer. RT-PCR analysis showed that P2X_7_(k)-specific primer pairs produced amplicons are present in striatal slices. The gel is 1.5%, 1× TBE buffer. A 100-bp DNA ladder (Fermentas, Vilnius, Lithuania) was used to identify PCR fragment sizes, and the amplification of 18s RNA was used as an internal control. C. Changes in mRNA expression levels of P2X_7_(k) variant in the striatum obtained from P2X_7 _receptor wild-type mice after *in vitro *rotenone pretreatment. Quantitative SYBR Green real-time PCR was performed using specific primers. The experiments were repeated twice with similar results. The expression level of the P2X_7_(k) isoform was normalized to the expression level of the distinct housekeeping gene 18S rRNA. Data are displayed as the means ± S.E.M. Asterisks indicate significant differences from the corresponding control (**P < 0.01, Student's t-test). D, E, F. Tissue contents of ATP, ADP and AMP, expressed in nmol/mg protein (n = 5-8). G. For the calculation of energy charge (EC) see Materials and Methods. * *P *< 0.05, ** *P *< 0.01, significance vs. control.

Striatal slices of WT mice were preincubated *in vitro *with the irreversible complex I inhibitor, rotenone (10 μM), for 60 min. Changes in the levels of mRNA transcripts of the P2X_7 _and P2X_4 _receptor subunits were measured using real-time RT-PCR. Gene expression level was normalized to the expression level of the 18S rRNA reference gene. Quantitative real-time PCR measurements revealed that the mRNA expression of the P2X_7 _receptor was up-regulated to 1.42 ± 0.12 of the corresponding control values (Figure 5A, n = 4, P < 0.05). In contrast, the mRNA level of the P2X_4 _receptor showed a decreasing tendency after rotenone treatment that did not reach the significance threshold (Figure 5A, 0.52 ± 0.26 of the corresponding control values; n = 4, P > 0.05).

A recent study has shown that, among the ten identified splice variants of P2X_7 _receptor subtypes, the P2X_7_(k) isoform has fully functional properties that contribute to the diversity of P2X_7 _receptor signaling in rodents [6]. We examined the mRNA expression of this splice variant using RT-PCR in control and 1 μM MPTP-treated (24 h) PC12 cells and in striatal slices of WT mice with and without a 10 μM *in vitro *rotenone pretreatment (60 min). We used a forward primer specific for exon 1' of the P2X_7_(k) variant with a common antisense primer in exon 7. The P2X_7_(k)-specific primer pairs produced amplicons in both control and rotenone-treated striatal slices but not in untreated and MPTP-treated PC12 cells (Figure 5B).

Next, we performed a quantitative identification of the expression of the P2X_7_(k) variant mRNA in striatal slices of WT mice. Real-time PCR analyses revealed that the mRNA expression of the P2X_7_(k) variants was down-regulated to 0.32 ± 0.07 of the corresponding control values, which were established as 1 and normalized to 18S reference rRNA, in response to a 60-min *in vitro *rotenone (10 μM) pretreatment (Figure 5C, n = 3, P < 0.01).

The basal ATP levels in the substantia nigra were significantly higher, but the ADP levels were significantly lower in WT mice than in the P2X_7_-/- littermates (Figures 5D, E). When the substantia nigral preparations, which contained the cell bodies of dopaminergic neurons that project to the striatum, were preincubated *in vitro *with rotenone (10 μM) for 60 min, the ATP content was significantly decreased in both WT and P2X_7_-/- mice (Figure 5D). The same treatment elicited a significant depletion of ATP content in the striatum of P2X_7_-/- but not in WT mice (Figure 5D). This decrease in ATP content by rotenone was paralleled with an increase in ADP level in the substantia nigra of WT animals (Figure 5E), but similar changes were not observed in the substantia nigra of P2X_7_-/- animals or in the striatum of either genotype (Figure 5E). AMP levels and the energy charge (EC) were not significantly altered in the substantia nigra and the striatum either by genetic deletion of P2X_7 _receptors or rotenone treatment (Figures 5F-G).

**Figure 6:**
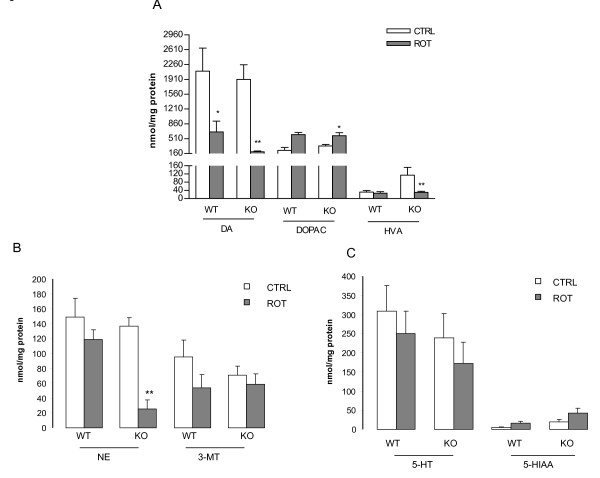
**DA and its metabolites in striatal slices after rotenone treatment in wild-type (WT) and P2X_7 _receptor knockout (KO) mice**. Samples were analyzed using HPLC, and the amount of (A) dopamine (DA) 3,4-dihydroxyphenylacetic acid (DOPAC), homovanillic acid (HVA), (B) norepinephrine, (NE), 3-methoxityramine (3-MT) and (C) 5-HT and 5-hydroxyindoleacetic acid (5-HIAA) is expressed in nmol/mg protein (n = 5-8). Asterisks indicate significant differences from the corresponding control (*P < 0.05, ** *P *< 0.01).

**Figure 7:**
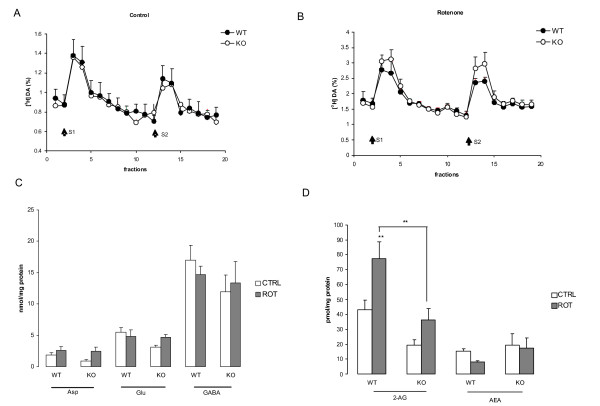
**The effect of *in vitro *rotenone pretreatment on EFS-evoked [^3^H]DA release and on the levels of glutamate (Glu), aspartate (Asp), GABA and of the endocannabinoid 2-AG and anandamide (AEA) in wild-type (WT) and P2X_7 _receptor knockout (KO) mice**. A, B. Slices were perfused with Krebs solution and subjected to electrical field stimulation (25 V, 1 msec, 2 Hz, 240 shocks) during the 3^rd ^and 13^th ^sample collection period (S1, S2) after a 60-min preincubation with rotenone (10 μM, B) or Krebs' solution (A). [^3^H]DA release is expressed as fractional release (FR%, for calculation see Materials and methods) as a function of time (n = 5-8). C, D. Samples were analyzed using HPLC, and the results are expressed in nmol/mg protein (C) or in pmol/mg protein (D) (n = 5-8). Asterisks indicate significant differences from the corresponding control or between WT and KO mice as indicated (** P < 0.01).

Rotenone treatment depleted striatal DA content in both WT and P2X_7_-/- mice, and the amount of DA metabolite DOPAC was significantly increased in the P2X_7_-/- group compared to saline treatment (*P *< 0.05, *P *< 0.01, Figure 6A). In slices derived from P2X_7_-/- mice, rotenone treatment also depleted the NE content (*P *< 0.01, Figure 7B). The amounts of 3-MT, 5-HT and 5-HIAA were insignificantly changed either by rotenone treatment or by the genetic deletion of P2X_7 _receptors (Figures 6B, C).

After loading striatal slices with [^3^H]DA, radioactivity uptake was 55996 ± 9346 Bq/g (n = 8) in WT mice and 71115 ± 15573 Bq/g (n = 4) in P2X_7_-/- mice, which were not significantly different from each other (*P *>0.05). Low frequency EFS (25 V, 2 Hz, 240 shocks) was used to mimic neuronal activity. This stimulation elicited a rapid and reproducible efflux of [^3^H]DA in striatal slices obtained from the WT and P2X_7_-/- groups (Figure 7A). Rotenone treatment did not change radioactivity uptake in the WT group (96084 ± 26879 Bq/g, n = 4; *P *> 0.05) and in the P2X_7_-/- group (42072 ± 6569 Bq/g, n = 8; *P *> 0.05), whereas electrical field stimulation evoked [^3^H]DA release also remained unchanged in WT and P2X_7_-/- mice (Figure 7B). These results imply that, despite the considerable depletion of endogenous DA content, striatal slices accumulate and release exogenous dopamine after rotenone treatment in response to neuronal activity, and the genetic deletion of P2X_7 _receptors did not alter this ability of dopaminergic nerve terminals.

The levels of the excitatory amino acids, glutamate and aspartate, and of the inhibitory amino acid, GABA, in the striatum did not change following *in vitro *rotenone treatment and was similar in WT and P2X_7_-/- animals (Figure 7C). In contrast, the content of the endocannabinoid, 2-arachidonoyl glycerol (2-AG), but not anandamide (AEA), was markedly elevated after rotenone treatment in the WT mice (Figure 7D). Although rotenone treatment also tended to increase 2-AG levels in the P2X_7_-/- mice, the elevation was much lower than in the presence of P2X_7 _receptors (Figure 7D).

## Discussion

The main objective of our study was to examine the role of the P2X_7 _receptor in various PD models. We demonstrated that the mRNA encoding the P2X_7 _receptor was up-regulated in animal models of PD. However, our results conflict with the concept that treatment with P2X_7 _receptor antagonists can alleviate the progress of PD.

In agreement with previous studies [33-35], rotenone treatment elicited a concentration-dependent decline in cell viability of PC12 cells and primary dopaminergic neurons, as measured using the MTT and LDH assays. This effect was also obvious in morphological signs and was significantly reversed by the antiparkinsonian drug, L-deprenyl. MPTP also decreased cell viability in PC12 cells in a concentration-dependent and deprenyl-sensitive manner, as measured with the MTT assay, and caused an increase the amount or released LDH in 10 μM concentration. However, a lower concentration (1 μM) of MPTP, did not elicit significant effect in PC12 cells and even increased cell viability in primary dopaminergic neurons. This latter finding is not surprising, however, because a transient proliferative effect of MPTP has been reported in PC12 cells after a 24 h incubation, which is dependent on the MEK1 pathway [36].

We used two P2X_7 _receptor antagonists in this model, BBG, which is selective for P2X_7 _receptors in the concentrations used here [37], and AZ10606120, which is a potent negative allosteric modulator of P2X_7 _receptors with a nanomolar IC_50 _value [38]. Both compounds were found to confer protection against MPTP treatment in both MTT and LDH assays and against rotenone-induced decrease in cell viability of PC12 cells when viability was measured using the LDH assay. Because the stimulation of P2X_7 _receptors leads to cell death in different cell types [32,39], these results imply that P2X_7 _receptors were endogenously activated by the released ATP, and thereby contributed to toxicity elicited by mitochondrial toxins.

In contrast, we could not detect a protective effect against rotenone of either BBG or AZ10606120, when cell viability was measured using the MTT assay in PC12 cells, and when primary midbrain culture was assessed. PC12 is a pheochromocytoma cell line able to synthesize catecholamines, whereas primary midbrain culture is a mixed population consisting of glia and MAP^+ ^neurons, including TH^+ ^immunopositive dopaminergic neurons. Therefore, one possible explanation to the lack of protective effect of P2X_7 _receptor antagonists in the primary culture is the presence of glia, which also express P2X_7 _receptors and, which might also influence the process leading to neuronal death. This assumption is supported by further observations of the present study using in vivo MPTP treatment and in vitro striatal slices. In fact we could not detect protective effect of genetic deletion of P2X7 receptors in those PD models, where the synaptic organization and its environment provided by the glia was retained.

The up-regulation of P2X_7 _and P2X_4 _receptors at the mRNA level was also observed in the substantia nigra and striatum after the *in vivo *treatment with the dopaminergic neurotoxin, MPTP. To examine whether these changes were also translated to protein synthesis, we examined the distribution of P2X_7 _receptor immunoreactivity in the striatum in saline- and MPTP-treated WT mice. As expected, P2X_7 _receptor immunofluorescence was localized to microglia in the striatum of WT mice. However, no visible change in P2X_7 _receptor immunostaining was observed after *in vivo *MPTP treatment. This result is in agreement with the findings of Amadio et al. [29], who found an unchanged P2X_7 _protein level after 6-OHDA denervation in the rat striatum. Although the precise cell type specific localization of P2X_7 _receptors requires further investigation, these findings imply that activity-dependent changes in the level of mRNA encoding P2X_7 _receptors is not accompanied with similar changes in protein synthesis, or that they are compensated by counter-regulatory events.

When mice were pretreated *in vivo *with MPTP, a dose-dependent and severe depletion of endogenous DA in the striatum was observed as a consequence of the ongoing degeneration of dopaminergic nerve terminals. Although basal dopamine levels were higher in the striata of P2X_7_-/- mice, MPTP treatment elicited a similar reduction in DA content in the WT mice. Moreover, after the genetic deletion of this receptor subtype, a reduction in NE content was also manifest at the lower dose of MPTP treatment. This result indicates an extension of neurodegeneration in the absence of P2X_7 _receptors, although endogenous 5-HT levels were relatively preserved. To test the effect of the pharmacological inhibition of P2X_7 _receptors, we utilized BBG, a P2X_7 _receptor antagonist that penetrates the blood brain barrier, and is efficacious in animal models of spinal cord injury [17,18] as well as Huntington's disease [20] in the dose applied. Nevertheless, treatment with BBG did not afford neuroprotection in our hands, at least using an acute treatment protocol.

It has been established previously that the pathological hallmarks of PD can be reproduced *in vivo *with the sub-chronic application of the irreversible mitochondrial complex I inhibitor, rotenone [40-42], or with the *in vitro *pretreatment of striatal slices with rotenone [33] in rats. Our findings in mice confirm and extend these data, i.e., the depletion of endogenous DA levels in the striatum concomitant with a decrease in endogenous ATP levels in the striatum and substantia nigra. Interestingly, a more pronounced ATP depletion was found in the substantia nigra than in the striatum after rotenone treatment, which corroborates with the well-known vulnerability of this brain area to mitochondrial toxins. Similar to the results seen after *in vivo *MPTP treatment however, genetic deletion of P2X_7 _receptors did not reverse these changes but exacerbated the damage and elicited a further drop of endogenous ATP and NE contents. Moreover, basal ATP levels were substantially lower in the substantia nigra of P2X_7_-/- mice. Taken together, P2X_7 _receptor deficiency did not ameliorate the effects of dopaminergic neurotoxins, rather these effects were potentiated.

Although these findings are somewhat unexpected in light of the regulation of excitotoxic and neuroinflammatory pathways by P2X_7 _receptors [2,3,43,44], there are several explanations for the lack of neuroprotection following genetic deletion and pharmacological antagonism of P2X_7 _receptors.

It is well known that results generated using knock-out mice must be treated with caution because compensatory mechanisms may occur. Our results clearly demonstrated that mRNA encoding the P2X_4 _receptor was up-regulated in the striatum of P2X_7_-/- mice compared to the WT group, and the expression levels of the P2X_7 _and P2X_4 _receptors were similarly elevated after MPTP treatment in PC12 cells. However, a similar change in P2X_4 _mRNA level was not observed in the rotenone-treated striatum. P2X_4 _and P2X_7 _receptors are frequently co-expressed [4] in particular on microglia, which is the cell type that governs neuroinflammation. Weinhold et al. [45] found that in alveolar epithelial cells, the expression of P2X_4 _receptors was strongly increased after P2X_7 _down-regulation, which indicates crosstalk and feedback signaling between these two receptors. Taking into account that the affinity of ATP for P2X_4 _receptors is much higher than for P2X_7 _receptors [5], the P2X_4 _receptor is a strong candidate to compensate the actions mediated by P2X_7 _receptors in cases of genetic deletion or pharmacological blockade.

Another possible interpretation of the lack of neuroprotection following the deletion of P2X_7 _receptors is the existence of splice variants, which partly retain their functionality in the CNS in different P2X_7 _knockout mice lines [6]. So far ten splice variants of the P2X_7_ receptor (P2X_7_(b) through P2X_7_(k)) have been described [6,46,47]. However, out of them only one, the recently identified P2X_7_(k) [6] has been found to be fully functional. Therefore, we have examined the mRNA expression of this variant in PC12 cells and in the rotenone treated rat striatum. The transcript encoding the P2X_7_(k) variant was expressed in the WT striatum, but its expression level was decreased after rotenone treatment, which does not support its involvement as a compensatory receptor.

A third, most intriguing possibility is that that activation of P2X_7 _receptors may also mediate neuroprotective effects, which could counterbalance its deleterious actions. The activation of P2X_7 _receptors in astrocytes enhances P2Y_2 _mRNA expression [48], which can trigger neuroprotective pathways through the induction of genes for anti-apoptotic factors, neurotrophins, growth factors and neuropeptides [49]. Moreover, recent studies have shown that the P2X_7 _nucleotide receptor is coupled to GSK-3 inhibition and confers neuroprotection in cerebellar granule neurons [50,51]. P2X_7 _receptors also play important roles in the synthesis and subsequent release of endocannabinoids from activated microglia and astrocytes [15,16]. The activation of CB1 and CB2 receptors by endocannabinoids mediates neuroprotection in different pathological models [52-54], including animal models of PD [55,56]. Our data indicate that this endogenous protective mechanism was activated after an *in vitro *rotenone treatment because an increased level of 2-AG was detected in the striatum. In contrast, elevation of 2-AG levels was absent in the striatum of P2X_7_-/- mice, which may lead to the alleviation of the 2-AG-mediated neuroprotective action.

Finally, an important factor that might influence whether beneficial or deleterious actions mediated by P2X_7 _receptors predominate, is the age of animals. In support of this idea, Jimenez et al. [57] found that at early ages (4-6 months), activated microglia are restricted to amyloid deposits and are characterized by the absence of cytotoxic factors using a transgenic mouse model of Alzheimer's disease. In contrast, at older ages, microglial activation shows a classic phenotype with the production of cytotoxic factors. Taking into account the governing role of P2X_7 _receptors in the activation and proliferation of microglia and in microglia-mediated death of neurons [2,12], the relatively young age of the mice used in our experiments may also explain the lack of protection by P2X_7 _inhibition.

## Conclusions

The results of experiments in different PD models do not indicate a consistent protective effect of deficiency or inhibition of the purinergic receptor subtype P2X_7_. Moreover, certain neurochemical alterations characteristic to PD were exacerbated after MPTP or rotenone treatment in P2X_7 _deficient mice, with a concomitant decline in the accumulation of the neuroprotective endocannabinoid 2-AG. We conclude that P2X_7 _receptor deficiency or inhibition does not promote the survival of dopaminergic neurons in an *in vivo *or *in vitro *models of PD and supports the view that P2X_7 _receptors play a double-faced role in different cellular cascades that lead to neuronal death.

## Methods

### Animals

All studies were conducted in accordance with the principles and procedures outlined in the NIH Guide for the Care and Use of Laboratory animals and were approved by the local Animal Care Committee of the Institute of Experimental Medicine (Budapest, Hungary, ref. No. 22.1/3671/003/2008). Two to 3-month old male wild-type (WT) and P2X_7 _receptor knockout mice (P2X_7_−/−) were housed in a light- (12 h on, 12 h off) and temperature-controlled room with food and water available *ad libitum*. All experiments were performed during the light phase between 7:30 am and 3:30 pm. Homozygous P2X_7 _receptor WT mice were bred on a background of C57Bl/6J. The original breeding pairs of P2X_7_R−/− mice were kindly supplied by Christopher Gabel from Pfizer, Inc. (Groton CT, USA). The animals contained the DNA construct P2X_7_-F1 (5'-CGGCGTGCGTTTTGACATCCT-3') and P2X_7_-R2 (5'-AGGGCCCTGCGGTTCTC-3'), previously shown to delete the P2X_7 _receptor [58]. Genomic DNA was isolated from the tails of WT and P2X_7_−/− animals, and the genotypes were confirmed by PCR analysis. Animals were randomly assigned to experimental clusters of 6-8 mice in each group. All animals were given intraperitoneal (i.p.) injections of sterile saline (0.9% NaCl) or 4 × 10-20 mg/kg MPTP, separated by 2 h. Treatment with the P2X_7 _receptor antagonist, Brilliant blue G (BBG, 50 mg/kg i.p), was administered 18 h before the first MPTP injection. Mice were euthanized by decapitation 72 h after the last treatment. The striatum was collected, frozen on dry ice and stored at −70°C until homogenization. Other mice were treated *in vitro*. After decapitation, the striatum was removed and sliced into 400- μm-thick sections, and the slices were incubated with 10 μM rotenone for 60 min in Krebs' solution kept at 37°C and bubbled with a mixture of 95% O_2 _+ 5% CO_2 _(see below).

### Cell culture

#### PC12 cells

PC12 cells were grown in Dulbecco's Modified Eagle's Medium supplemented with 10% heat-inactivated fetal bovine serum. The cultures were maintained in a humidified atmosphere containing 5% CO_2 _at 37°C. For experiments assessing the effect of drugs on rotenone- or MPTP-induced cell death, PC12 cells were seeded on 96-well plates in a density of 5000 cells/well, preincubated with 10 and 100 nM of L-deprenyl, AZ10606120 or BBG for 1 h and then treated with rotenone (0.01-30 μM) or MPTP (0.01-30 μM). The incubation was then continued for 20 h at 37°C.

#### Cultures of ventral midbrain cells

Timed pregnant C57BL/6 mice were deeply anesthetized with an i.p injection of a xylazine-ketamine mixture on days 12-13.5 after vaginal plug identification. The embryos (E12 - E13.5) were aseptically dissected and rinsed with a Ca^2+^-free PBS (pH 7.4) containing 40 μg/ml gentamicin (Sanofi-Aventis) and 250 μg/ml fungizone (Sigma, Hungary). The brains were rapidly removed and the ventral mesencephali were dissected. Tissue pieces were incubated in a trypsin solution (0.5% trypsin (w/v; Sigma, Hungary) in PBS containing 50 μg/ml DNAse I (Sigma, Hungary) for 20 min at room temperature. After incubation, the tissue pieces were transferred onto a nylon mesh (pore diameter 43 μm) fixed to the bottom of a polypropylene tube and triturated within the tube in 10% FCS in Minimum Essential Medium (MEM-FCS) (Sigma, Hungary) using a Pasteur pipette. The suspension of single cells streamed through the mesh was collected (approx. 2 ml/embryo). After counting in a Bürker chamber, the cells were plated in MEM-FCS onto poly-L-lysine coated 96-well culture plates (105 cells/100 μl/well; Falcon; BD) or onto poly-L-lysine coated glass cover slips (d = 12 mm; 5 × 105/500 μl/well) in 4-well plates. After adhesion of cells, the medium was changed and the cells were maintained in DMEM/F12 (1/1) supplemented with B27 (Invitrogen). The medium was changed every second day and was supplemented with 20 ng/ml BDNF (Invitrogen) from the second day. Cultures were treated on days 6-7 *in vitro *with L-deprenyl, AZ10606120 or BBG and then with 1 μM rotenone or 1 μM MPTP as described above.

### Quantitative real-time PCR experiments

The striata and the PC12 cells were lysed and homogenized in Trizol. Total RNA was isolated from the cell lysates using the RNeasy Lipid Tissue Mini Kit (Qiagen) according to the manufacturer's instructions. The purified RNA was reverse-transcribed with a RevertAid First Strand cDNA Synthesis Kit (Fermentas, Vilnius, Lithuania) as described in our previous study [59]. Briefly, the cDNA samples were prepared by reverse transcribing 2 μg of total RNA using 1 μl of RevertAid H Minus M-MuLV reverse transcriptase in a mixture containing 5 μl of 5× reaction buffer, 1 μl random hexamer primer (10 pmol/μl), 1 μl of RiboLock™ RNase Inhibitor (20 u/μl) and 2 μl of 10 mM dNTP mix, which was brought up to a final volume of 20 μl with 0.1% diethylpyrocarbonate-treated distilled water. The reverse-transcription reaction was performed at 70°C for 5 min and was followed by incubation at 25°C for 5 min. The synthesis was continued for 10 min at 25°C and was followed by 60 min at 42°C. The samples were finally stored at −20°C. The expression levels of the target genes were determined from the cDNA samples using quantitative real-time PCR (Rotor-Gene 3000; Corbett Research, Sydney, Australia). Real-time PCR was performed according to standard protocols using the LightCycler DNA Master SYBR Green I Kit (Roche, Indianapolis, IN, USA). PCR conditions were optimized for primers, templates and MgCl_2_. The PCR cycling protocol was set to the following parameters: initial denaturation, 95°C for 10 min; cycling, 95°C for 20 s, 59°C for 20 s, 72°C for 20 s; 45 cycles. All PCR primers were based on a previous study [60] with the following sequences: P2X_4 _receptor forward: 5'-ATC GTC ACC GTG AAC CAG ACA CA, reverse: 3'-CCA CGA TTG TGC CAA GAC GGA AT P2X_7 _receptor forward: 5'-CCA CAA CTA CAC CAC GAG AAA C, reverse: 3'-ACT TCT TGG CCC TTG ACA TCT T; 18S forward: 5'-GTA ACC CGT TGA ACC CCA TT, reverse: 3'-CCA TCC AAT CGG TAG TAG CG. Primers for the amplification of the P2X_7_(k) variant cDNAs were based on the study of Nicke et al. [6] and were the following: P2X_7 _receptor 1' exon-specific forward primer 5' GCC CGT GAG CCA CTT ATG C-3' and reverse primer in exon 7 5'-TCT GTA AAG TTC TCT CCT GC-3'. The conditions for amplification in this case were as follows: 95°C for 10 min, initial denaturation at 94°C for 30 s, 59°C for 30 s, and 72°C for 30 s for 45 cycles with a final extension at 72°C for 7 min. To ensure the specificity of the reaction and accurate quantification, a melting curve analysis was performed after each reaction, which confirmed the lack of primer-dimer artifacts or contamination in all cases. All ΔCt values were calculated using the Rotor-Gene 6 software (Corbett Research, Sydney, Australia). Expression levels of the target genes were normalized to the reference gene, 18S rRNA. The target gene and the reference gene were measured together within the same experiment. To compare the expression levels of target genes between different experimental groups, the efficiency calibrated model of Pfaffl [61] was applied. Differences in gene expression were considered significant when the *P *level was < 0.05. Data are presented as the mean normalized expression ratio ± SEM.

### RT-PCR amplification of P2X_7_(k) variant mRNAs

The striata and the PC12 cells were lysed and homogenized in Trizol. Total RNA samples were isolated and purified from the cell lysates using the RNeasy Lipid Tissue Mini Kit (Qiagen) according to the manufacturer's instructions. RNA (2 μl) was reverse-transcribed with the RevertAid First Strand cDNA Synthesis Kit (Fermentas, Vilnius, Lithuania) as described in our previous study [59]. Primers for amplification of the P2X_7_(k) variant cDNAs were subjected to RT-PCR amplification under the following conditions: 35 cycles, initial denaturation at 94°C for 30 s, 59°C for 30 s, and 72°C for 30 s for 35 cycles with a final extension at 72°C for 7 min.

### Fluorescence microscopy

Primary cultures grown on microscopic cover slips for 8 days were rinsed with PBS and fixed in 4% paraformaldehyde (w/v in PBS) for 40 min at room temperature. After washing with PBS, the cultures were treated with 0.1% TritonX100 in PBS for 5 min and non-specific binding sites were blocked by a 2-hour incubation with 3% donkey and normal horse sera (Jackson ImmunoResearch Europe Ltd. Newmarket, Suffolk, UK) in 0.1 M phosphate buffer (PB, pH 7.4). Cells were incubated with a mixture of MAP2 (1:1000; polyclonal rabbit; Abcam, Cambridge, UK) and tyrosine hydroxylase (1:500; monoclonal mouse; Chemicon, Billerica, MA, USA) primary antibodies at 4°C overnight. For control experiments, the primary antibodies were omitted. Biotinylated anti-mouse IgG (Vector Laboratories; Burlingame, CA, USA) and Alexa Fluor 594-conjugated anti-rabbit IgG (Molecular Probes/Invitrogen) (1:1000) were applied as secondary antibodies for 2 h at room temperature. Streptavidin-FITC (1:1000) in PB was applied for 2 h. Stained cultures were mounted in Mowiol 4-88 (Polysciensce Inc, Warrington, PA, US) containing Hoechst nuclear dye and investigated with an Axiovert 200 M microscope and the AxioVision 4.8 program (Zeiss, Jena Germany).

### Laser scanning confocal microscopy for the investigation of P2X_7 _receptor localization in striatal sections

After removing the brain, the striatum was removed and fixed for 2 × 1 h in 4% paraformaldehyde in 0.1 M phosphate-buffered saline (pH 7.4). Thirty-micron sections were cut using a vibrating microtome (VT1000S; Leica Microsystems, Milton Keynes, UK). The sections were washed, kept in 0.1 M phosphate-buffered saline and then transferred to 30% sucrose in PBS at 4°C until they sank. Freeze-thawing in liquid nitrogen was applied to increase the penetration of the antisera used for immunostaining. The sections were incubated first in the mixture of 3-3% donkey and normal horse sera (Jackson ImmunoResearch Europe Ltd. Newmarket, Suffolk, UK) in 0.1 M phosphate buffer pH 7.4 for 2 h, and then in the 1:200 dilution of the P2X_7 _polyclonal antibody (Santa Cruz Biotechnology, Inc. Santa Cruz, CA, USA) overnight at 4°C. After thorough washing, fluorescein-labeled GSL I - isolectin B4 (Vector Laboratories; Burlingame, CA, USA) in a 1:100 dilution and 1:400 DyLight 549 AffiniPure Donkey Anti-Goat IgG (Jackson ImmunoResearch) were applied overnight. Sections incubated without primary antibodies served as controls. After final washes in PBS, the sections were transferred onto microscopic slides and mounted in Vectashield (Vector Laboratories; Burlingame, CA, USA). Confocal microscopic images were scanned with a Nikon A1R microscope equipped with NIS-Elements C software. Images were edited and the brightness and contrast were adjusted if necessary using Adobe Photoshop CS3 (San Jose, CA, USA).

### Cell viability assays

Cell viability was assessed using the colorimetric reagent, 3-[4,5-dimethylthiazol-2-yl]-2,5-diphenyl tetrazolium bromide (MTT). A stock solution of the dye was prepared, filter-sterilized and stored at -20°C. After MPTP or rotenone treatment in 96-well plates, 5 mg/ml MTT was added to each well, and the incubation was continued for another 4 h. The converted dye was solubilized with acidic isopropanol (0.04 M HCl in absolute isopropanol). Reduced MTT was measured at a wavelength of 570 nm.

As another indicator of cell viability, lactate dehydrogenase (LDH, E.C. 1.1.1.27) was also assayed in experiments performed on PC12 cells. LDH activity that had been released into the media was evaluated using the CytoTox96 nonradioactive assay kit (Promega, Madison, WI, USA) according to the manufacturer's instructions. The LDH activity was quantified by measuring the wavelength absorbance at 490 nm with a Perkin-Elmer 1420 Multilabel counter. The released LDH activity is expressed as a percentage of total LDH activity, which was determined after freeze-thaw lysing of the cells.

### HPLC determination of adenine nucleotides, monoamines, amino acids and endocannabinoids

Animals were sacrificed by decapitation, and the striatum or the substantia nigra was dissected on ice under a stereomicroscope. In the case of *in vivo *MPTP-treated animals, after the preparation of the striatum, the native tissue was frozen in liquid nitrogen. In the other experiments, striatal slices or substantia nigra preparations were frozen after a 1 h *in vitro *pre-incubation with rotenone (10 μM). The weighed frozen tissue was homogenized in an appropriate volume of ice-cold 0.1 M perchloric acid that contained theophylline (as an internal standard) in a 10 nmol/ml concentration and 0.5 mM sodium metabisulfite (antioxidant for biogenic amines). The suspension was centrifuged at 300 g (4500 rpm) for 10 min at 0-4°C. The excess perchlorate anion in the supernatant was removed by the addition of 2 M KOH in 7:3 ratios, respectively. The potassium perchlorate was removed by centrifugation as described above. The supernatant was kept at -20°C until analyzed. The pellet was saved for protein measurement according to Lowry et al. [62].

A Gilson liquid chromatographic System with 715-operation software (Gilson Medical Electronics Inc., Middletown, WI, USA) was used. Two delivery pumps (Model 305) and a programmable auto injector (Model 231 XL) were built in. The system was equipped with BAS CC-4 amperometric and Agilent 1100 variable wavelength detectors in a cascade line. For sample cleaning, a "trap-column" (15-25 μm Nucleosil C-18 (20 × 4.0)) was inserted into a loop position. The separations were performed on a 3 μm Discovery C18 HS (150 × 4.0 mm) analytical column. For the quantification of nucleotides and biogenic amines, a two-dimensional reversed-phase and an ion pair chromatographic separation were used [40]. After the elution of nucleotides, an ion pair-reversed-phase buffer at constant flow rate (0.8 ml/min) was applied from the 11th to 55th min of analysis. The detection of adenine nucleotides and internal standards (IS) was performed at 254 nm wavelengths by UV, and the biogenic amines were measured at a 0.73 V potential of electrochemical detection. The retention order of UV-detected compounds were as follows: adenosine 5'-triphosphate (ATP) 3.6 min, adenosine 5'-diphosphate (ADP) 6.1 min, adenosine 5'-monophosphate (AMP) 11.7 min and theophylline (IS) 21.2 min. The order of monoamines were norepinephrine (NE) 14.7 min, 3,4-dihydroxyphenylacetic acid (DOPAC) 16.8 min, 5-hydroxy indolacetic acid (5-HIAA) 19.3 min, dopamine (DA) 25.5 min, homovanillic acid (HVA) 26.8 min, 3-methoxytyramine (3-MT) 31.2 min and 5-hydroxytryptamine (5-HT) 39.3 min.

The separation of pre-column dansylated amino acids and endocannabinoids was performed with a gradient elution-working mode at ambient temperature. The mobile phase A consisted of 5/95 (v/v) 78/22 acetonitrile/methanol in 15 mM ammonium formate buffer, and the mobile phase B was composed of 90/10 (v/v 78/22) acetonitrile/methanol in ammonium formate buffer, pH 3.7. The mobile phase B increased linearly (at 0.11 min to 50% at 17 min 72% and at 26 min 100% and was run to 54 min), and the flow rate was 0.7 ml/min. The analytical and the trap column were equilibrated for 10 min, and the enrichment and clean-up procedures were started. Dansylated derivatives were detected using an absorbance detector (Agilent 1100) at a 319 nm wavelength. The retention times of dansylated compounds were as follows: aspartic acid (Asp) 10.3 min, glutamic acid (Glu) 11.4 min, gamma amino butyric acid (GABA) 16.2 min, AEA 48.5 min and 2-AG 50.5 min. Pre-column derivatization was performed by mixing 50 μl of dansyl chloride (prepared by dissolving 5 mg 5-(dimethylamino)naphthalene-1-sulfonyl chloride in acetonitrile daily and adding 50 μl of 2 M sodium carbonate, which contained norvaline as an internal standard in 20 μM with 25 μl of sample. After a 10-min reaction time at 60°C, the mixture was acidified with 25 μl of 6 M formic acid and injected onto the "trap-column".

The concentrations of separated compounds were calculated using a two-point calibration curve internal standard method: (Ai * f * B)/(C * Di * E) (Ai: Area of component; B: Sample volume; C: Injection volume; Di: Response factor of 1 pmol of standard; E: Protein content of sample; f: factor of Internal Standard (IS area in calibration/IS area in actual)). The data are expressed as nmol per mg protein or as pmol per mg protein.

### [^3^H]Dopamine release experiments

Mice were decapitated and the brain was quickly removed into an ice-cold Krebs' solution. The striatum was dissected out and sliced into 400-μm-thick sections with a McIlwain tissue chopper. The release experiments were performed as described previously [33,41]. Briefly, the slices were pretreated with rotenone (10 μM) for 60 min and then rinsed with a normal Krebs' solution. The slices were then incubated in 1 ml of Krebs' solution containing 5 μCi [^3^H]DA for 45 min, during which they were continuously gassed with a mixture of 95% O_2 _and 5% CO_2 _at 37°C. After incubation, the slices were rinsed and transferred to tissue chambers and perfused continuously with a modified Krebs solution (in mM: NaCl 113, KCl 4.7, CaCl_2 _2.5, KH_2_PO_4 _1.2, MgSO_4 _1.2, NaHCO_3 _25, Na_2_EDTA 0.03, ascorbic acid 0.3, and glucose 11.5) at a rate of 0.7 ml/min. After a 60-min pre-perfusion, 3-min samples were collected and assayed for [^3^H]DA.

Electrical field stimulation (EFS, S1, S2) was delivered by a Grass S88 stimulator twice, during the 3^rd ^and 13^th ^sample of the collection period, using platinum ring electrodes attached to the top and bottom of tissue chambers with the following parameters: 25 V, 1 msec, 2 Hz, 240 shocks. At the end of the experiment, the slices were homogenized in 0.5 ml of 10% trichloroacetic acid. A 0.5 ml aliquot of the superfusate and 0.1 ml of the tissue supernatant were added to 2 ml of scintillation cocktail (Ultima Gold, Packard). Tritium was measured with a Packard 1900 TR liquid scintillation counter using an internal standard and expressed as percentage of the amount of radioactivity in the tissue at the time of sample collection (fractional release, FR %). The tissue tritium uptake was determined as the sum release + the tissue content after the experiment and expressed in Bq/g.

### Materials

The following materials were used: brilliant blue G (BBG), L-deprenyl, DMSO, Dulbecco's Modified Eagle's Medium, fetal bovine serum, 1-methyl-4-phenyl-1,2,3,6-tetrahydropyridine (MPTP), rotenone, 3-[4,5-dimethylthiazol-2-yl]-2,5-diphenyl tetrazolium bromide (MTT), (all from Sigma-Aldrich, St. Louis, MO), AZ10606120 dihydrochloride (Tocris Bioscience, Ellisville, MO, USA). All solutions were freshly prepared on the day of use.

### Data analysis

All data are expressed as means ± S.E.M. of *n *observations. Statistical analyses were performed using an ANOVA followed by the Dunnett test (multiple comparisons) or Student's t-test (pair-wise comparisons). The log-rank test was used for the statistical evaluation of survival data. Graphpad Prism software was used for all statistical analyses. P values of less than 0.05 were considered statistically significant.

Adenylate energy charge (EC), introduced by Atkinson [63], was calculated accordingly:

The EC value represents the energy resources of a cell as a function of the concentration of nucleotides. A biological system is fully charged when ATP dominates over other adenine nucleotides, and the corresponding EC is close to one.

## List of abbreviations

AD: Alzheimer's disease; [^3^H]DA: [^3^H]dopamine; DMSO: dimethylsulfoxide; DOPAC: 3,4-dihydroxyphenylacetic acid; EC: energy charge; EFS: electrical field stimulation; 5-HIAA: 5-hydroxyindoleacetic acid; 5-HT: Serotonin; HVA: homovanillic acid; HD: Huntington's disease; LDH: lactate dehydrogenase; MAP2: microtubule-associated protein 2; 3-MT: 3-methoxytyramine; MPTP: 1-methyl-4-phenyl-1,2,3,6-tetrahydropyridine; MTT: 3-[4,5-dimethylthiazol-2-yl]-2,5-diphenyl tetrazolium bromide; NE: norepinephrine; PD: Parkinson's disease; PBS: phosphate buffered saline.

## Competing interests

The authors declare that they have no competing interests.

## Authors' contributions

ZH and CC performed the gene expression studies. ZH and FG maintained cell cultures and performed the cell viability assays. MB carried out the HPLC analyses. EM established the primary midbrain culture. AK performed the immunostaining experiments. ZH, MB, CC, FG, EM and AK drafted the manuscript. BS designed and coordinated the study and finalized the manuscript. All authors read and approved the final manuscript.
